# The role of transitional probabilities in word holistic processing

**DOI:** 10.1177/03010066241279932

**Published:** 2024-09-17

**Authors:** Paulo Ventura, Francisco Cruz, Alexandre Pereira

**Affiliations:** 37809 Universidade de Lisboa, Portugal; 70887 Universidade Lusófona de Humanidades e Tecnologias, Portugal

**Keywords:** visual words, holistic processing, composite task, transitional probabilities, bigrams

## Abstract

In recent years, increased attention has been devoted to visual word recognition under a perceptual expertise framework. Because the information required to identify words is distributed across the word, a holistic attentional strategy is optimal and develops with experience. It is, however, an open question the extent to which other information embedded in a word may contribute to word holistic processing, namely sublexical word properties. In the present research, we therefore explore the role of sublexical properties—specifically bigram transition probabilities—in this processing strategy. We used a common task in the holistic processing literature (i.e., composite task) and four-letter disyllabic words, where two of the bigrams reinforce the cohesiveness of each syllable and one of the bigrams reinforces the cohesiveness between the syllables. We found preliminary evidence of a role of these sublexical properties in word holistic processing.

Face perception is a highly challenging perceptual task. Faces share a global configuration, having the same set of features organized in one common structure (e.g., eyes above nose). Due to this, identifying a face requires the fast and accurate discrimination of fine-grained differences in these features (e.g., eye size) and their relationships (e.g., nose–mouth distance) through holistic processing (Diamond & Carey, 1986; Gauthier et al., 2003; Gauthier & Bukach, 2007; Rossion, 2013; Wong et al., 2009; Young et al., 1987). Holistic processing describes the attentional strategy of attending to the whole set of diagnostic facial features together, brought about by extensive expertise with faces (Chua et al., 2014; Richler et al., 2011a, 2012). Processing all parts of a face object together is an efficient strategy because its diagnostic features are spread across the entirety of the stimuli (Curby et al., 2024).

The conditions underlying the emergence of holistic processing—specifically, this need for intra-categorical discrimination and the role of expertise as promoting its accuracy—apply to other visual stimuli, and thus holistic processing may not be exclusive to faces. Individuals have extensive experience with written words, ubiquitous stimuli in human societies. Also, words are made up of a limited set of letters which makes the need for individuation of the words. Thus, words may also be processed holistically. The word composite effect supports this assertion, since it describes the tendency for the whole word to be attended, albeit only part of it is required for a task (i.e., in alphabetic and logographic scripts alike; Chen et al., 2013; Ventura et al., 2017; Wong et al., 2011, 2012). Prior studies have shown that words show a composite effect: When readers perform a same–different matching task on a target part of a word, performance is affected by the irrelevant part. Importantly, the word composite effect does not have its locus on processing stages associated with lower-level features. That is, the word composite effect emerges even when the word representations vary in their word features. Indeed, it is observed in written words varying in surface features (between-participants: courier, notera (simulating handwriting), alternating-cAsE), unaffected by NoVeLtY of the conﬁguration or by handwritten form (Ventura et al., 2017).

This composite effect is assessed using a composite task, in which participants are asked to indicate whether a matched syllable (e.g., left) in two consecutive dissyllabic words is the same or not. In this task, participants do not need to read nor to process the entirety of the word as there is an irrelevant syllable (e.g., right). Interestingly, the responses provided are influenced by the presence of the irrelevant syllable. When the response suggested by the irrelevant word part matches the correct response (i.e., pertaining to the relevant syllable), performance is better (e.g., same-response trials: LANE–LANE; different-response trials: LANE–COZY), relative to when the responses suggested by the relevant and irrelevant parts are in conflict (e.g., same-response trials: LANE–LADY, different-response trials: LANE–CONE). This effect of congruency is attenuated, however, when the syllables in the word are not aligned (e.g., one half of the word is displaced vertically, up or down). Although attenuated by misalignment, generic congruency effects may also be seen as evidence of holistic processing. In some earlier work with faces, a misaligned condition was not included and the congruency effect on aligned trials was used to index holistic processing (e.g., Richler, Gauthier, et al., 2008). However, later work showed that congruency effects that are not modulated by alignment can sometimes be observed in novices for strategic reasons (see Richler & Gauthier, 2014); that is not the case for our participants which are all expert readers.

As mentioned above, a holistic attentional strategy is optimal and thus develops through experience in processing expertise stimuli, including words. In the case of words, sublexical properties may also be important. In each writing system, there are combinations of characters more likely to occur than others. Thus, the probability of any letter in a sequence (e.g., in pairs—bigrams—or triads—trigrams) can be informed by those that precede or succeed it. And in the case of bigrams, this expected value is called bigram transitional probability (TP). In the word puro, <pu > corresponds to bigram 1, <ur > to bigram 2, and <ro > to bigram 3. A high TP means that one letter is more predictable when a given other is present. A high TP of bigram 1 and bigram 3 would strengthen the cohesiveness of the first and second half of a four-letter word while a high TP of bigram 2 would strengthen the connections between the two syllables. TPs are believed to be acquired through statistical rule learning, predicting reading capabilities (see, e.g., Frost et al., 2019). We defined joint bigram TP (three bigrams in four-letter words with two syllables: consonant–vowel–consonant–vowel) considering both the mean and number of words sharing a bigram, as well as their summed and mean frequency. These metrics allowed us to define high bigram TP words (e.g., “caro”) versus low bigram TP words (e.g., “ruga”).

Here, we investigated whether there are effects of bigram frequencies as a proxy for TP on the word composite effect. We expect to observe congruency effects with or without an interaction with alignment. The potential role of TP on holistic word processing can be inferred from how the former modulates the latter: if TPs are important in driving holistic processing in words, then we should obtain congruency effects with or without an interaction with alignment which would be modulated by bigram TP. The absence of such interactions could mean that TPs have no influence on holistic word processing. Considering that holistic processing describes the simultaneous processing of multiple components of a whole, the combined integration of multiple information could inform the likelihood of a correct identification—thus hinting that TP may be extracted when holistic processing takes place. Essentially, should the visual system capitalize on contextual information (of which TPs are an example), this information might influence the holistic processing of words.

## Method

### Participants

Using MorePower 6.0.4 (Campbell & Thompson, 2012) a sample size of 34 would be required to find a medium effect size (η_p_^2 ^= 0.2) at α = .05 and a power of 0.8 for a 2 × 2 × 2 repeated-measures analysis of variance (ANOVA).

All students enrolled in a psychology course in Faculdade de Psicologia of Universidade de Lisboa were invited to participate due to anticipated data exclusion considering our exclusion criteria (defined a priori but not preregistered, see below). Forty-seven participants took part in the experiment. Data from eight participants were excluded.

The study's protocol adhered to the guidelines of the Declaration of Helsinki and the Portuguese deontological regulation for Psychology and was approved by the Deontological Committee of Faculdade de Psicologia of Universidade de Lisboa. All participants provided verbal informed consent.

### Material

Words for each quartet were selected such that they differed in the probabilities of stimuli co-occurrences, more specifically bigram TP. In a quartet of words, for example, caro, pule, cale, puro, each word appeared both as study and test stimuli, and allowed for 16 trials considering the intersection of “same” versus “different” response and “congruent” versus “incongruent” response. For example, and with the left half as the relevant part (see Appendices A and B for all the trials for the quartet “caro, pule, cale, pule”):

**Table:** 

Same–congruent	caro-caro
Different–congruent	caro-pule
Same–incongruent	caro-cale
Different–incongruent	caro-puro

We started with all the possible consonant vowel.consonant vowel quartets in European Portuguese and computed the bigram properties (the number and mean number of words sharing bigrams as well as their summed and mean frequency) for all words in each possible quartet based on P-PAL's metrics; P-PAL is a lexical database for European Portuguese (Soares et al., 2018). The final stimuli were 20 quartets of four-letter (consonant–vowel, consonant–vowel) consonant vowel.consonant vowel Portuguese words, 10 of high bigram TP and 10 of low bigram TP words (see Appendices A and B).

The two sets of quartets were controlled for several important linguistic variables. All variables were computed (see Table 1) based on P-PAL's metrics (Soares et al., 2018). Namely, we controlled for: (a) occurrences (per million words), (b) Zipf value (i.e., logarithmic frequency metric; see van Heuven et al., 2014), (c) orthographic neighbors (i.e., existing words formed by replacing a letter, see Coltheart et al., 1977; both their number and respective mean frequency), (d) phonological neighbors (i.e., existing words formed by replacing one phoneme, see Luce & Pisoni, 1998; both their number and respective mean frequency). We then conducted Bayesian versions of the *t*-tests designed to attest the absence of differences in the quartets for the variables mentioned above. According to Lee and Wagenmakers (2014), Bayes factors can quantify the strength of evidence both against (anecdotal: 1 < BF ≤ 3; moderate: 3 < BF ≤ 10; strong: BF > 10) and in favor (anecdotal: 0.33 ≤ BF < 1; moderate: 0.1 ≤ BF < 0.33; strong: BF < 0.1) of a null hypothesis, whereas null hypothesis significant testing can only account for the former. We find anecdotal (occurrences per million words: BF = 0.42, number of orthographic neighbors: BF = 0.64, and number of phonological neighbors: BF = 0.65) to moderate (Zipf value: BF = 0.28, mean frequency of orthographic neighbors: BF = 0.32, and mean frequency of phonological neighbors: BF = 0.24) support against the existence of differences for the variables controlled for above.

**Table 1.** Characteristics of high versus low TP words.

	High TP	Low TP	*p*
Frequency per million	28.18	8.88	0.26
Frequency Zipf scale	3.0	2.8	0.58
Number of orthographic neighbors	12.8	10.83	0.09
Mean word frequency of orthographic neighbors	18.6	22.49	0.59
Number of phonological neighbors	13.3	11.33	0.21
Mean word frequency of phonological neighbors	20.0	21.0	0.92
Number of words sharing bigrams	85.1	70.8	0.10
Mean number of words sharing bigrams	28.37	23.58	0.10
Summed frequency of words sharing bigrams	6524.0	2206.2	0.04
Mean frequency of words sharing bigrams	69.8	27.9	0.05
Number of words sharing trigrams	8.4	7.3	0.06
Mean number of words sharing trigrams	4.2	3.7	0.06
Summed frequency of words sharing trigrams	226.7	219.7	0.94
Mean frequency of words sharing trigrams	24.6	22.9	0.84

To manipulate TP, we selected 80 words (20 quartets) based on their bigram properties. High and low bigram TP were defined based on the number and mean number of words that shared bigrams with the words and the frequency of their bigrams (both summed and mean). Accordingly, Bayes factors suggest that there are meaningful differences between high and low TP words in these metrics (be it anecdotal—mean per million word frequency of the four-letter words sharing the first, second, or third bigram with the stimuli: BF = 1.81—moderate—summed per million word frequency of these words: BF = 3.61—or strong—the number of four-letter words sharing the first, second or third bigram with the stimuli: BF = 5.85, and mean number of words sharing the same bigrams with the stimuli: BF = 5.85). Additionally, we controlled for all of these properties regarding to trigrams statistics (three letter co-occurences within the word, e.g., in the word puro, <pur > corresponds to trigram 1, and <uro > to trigram 2) such that high and low TP words did not differ in the number and mean number of words with shared trigrams, nor on the frequency and mean frequency of the trigrams. There is anecdotal (number of four-letter words sharing the first or second trigram with the stimulus: BF = 0.69, mean number of four-letter words sharing the first or second trigram with the stimulus: BF = 0.69) to moderate (summed per million frequency of these words: BF = 0.24, mean per million frequency of these words: BF = 0.24) evidence against the existence of these differences.

### Procedure

**Figure 1.**
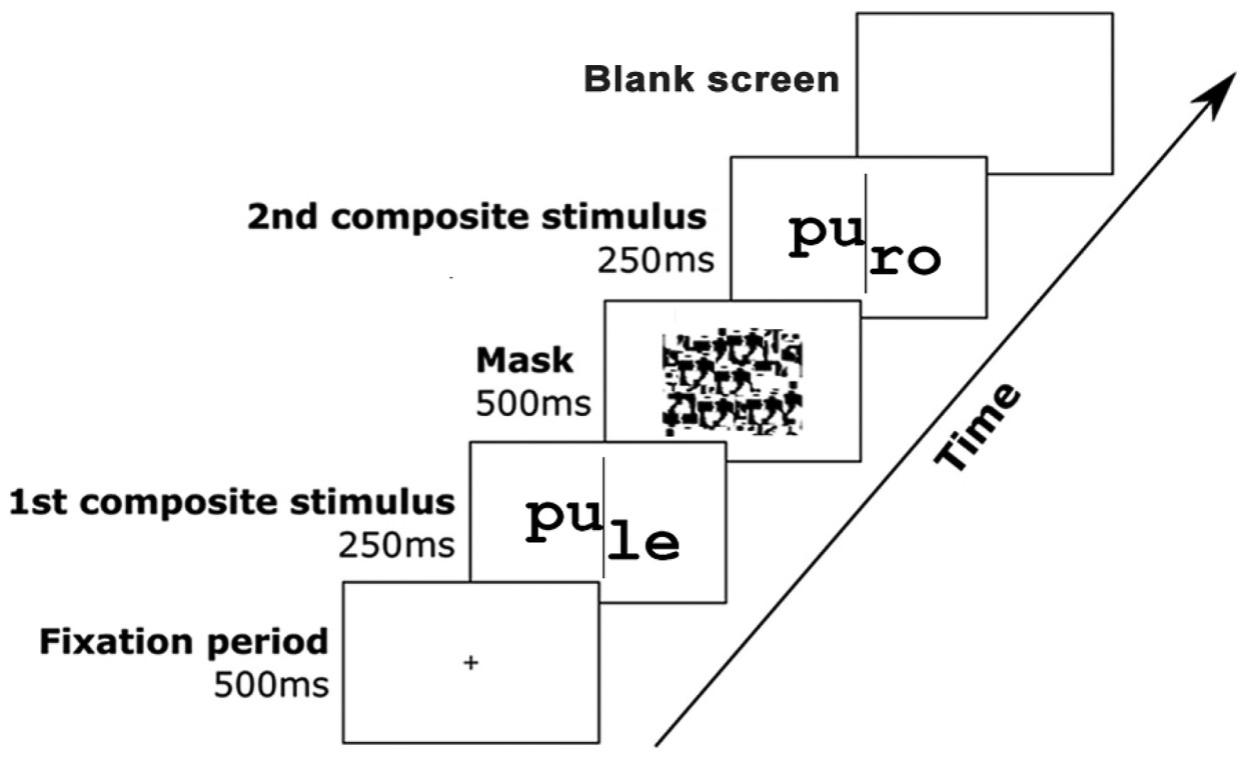
Events in a trial.

Stimuli were presented on a 17-in. CRT monitor, and E-Prime 2.0 was used to control stimulus presentation and response time (RT) recording (cf. Figure 1). Two words were shown sequentially during 250 ms and separated by a 500-ms mask. Each word was divided into the left and right halves by a vertical line. Within each set, the left and right halves of each word were interchanged to create the four words resulting from the orthogonal manipulation of response (same; different) and congruency (congruent; incongruent): for example, caro, pule, cale, puro. Each word appeared both as study and test stimuli. Thus, the same distractor parts were used in different conditions (congruent and incongruent). Participants performed eight blocks of trials (four aligned; four misaligned; block- and trial-order randomized), each with 80 trials. As shown in Figure 1, in the misaligned condition, the right half of the word was moved down by 100 pixels (visual degree angle 6.71° × 3.04°; visual degree angle for aligned condition 6.71° × 1.77°). Before the experimental trials, participants performed 16 computerized practice trials with different stimuli.

RTs were collected from the onset of the test word, and participants had to judge if the target parts (always the left halves) of the two words were identical or not by pressing the “1” or “2” key (with a green or red label) as quickly and accurately as possible, while ignoring the other, irrelevant part. After 2.5 s or upon response, the stimulus disappeared, and a blank screen appeared for 500 ms before the beginning of the next trial. The task adopted the complete version of the composite paradigm (Richler & Gauthier, 2014). In this version, one can define four types of trial (based on the crossing of correct response—same vs. different—and congruency with the irrelevant part—congruent vs. incongruent): same–congruent, same–incongruent, different–congruent, and different–incongruent. In half of the trials, the target and irrelevant parts are aligned with each other and in the other half they are misaligned. Holistic processing is typically inferred from an interaction between alignment and congruency (e.g., Richler et al., 2011b): performance in congruent trials is attenuated relative to incongruent trials especially when the parts are misaligned as opposed to aligned. Although attenuated by misalignment, generic congruency effects can also be evidence of holistic processing (e.g., Richler, Gauthier, et al., 2008).

## Results

Four outlier participants, with mean RT > 2.5 SD from the group mean were identified and removed from further analysis. Four participants had low sensitivity (mean d′ < 0) across all conditions and were dropped from the analysis. Thus, the results of 39 participants were analyzed. All exclusion criteria were determined a priori although not preregistered.

### Sensitivity

The statistic d′ (“d-prime”) is a measure of sensitivity considering the difference between hits and false alarms. However, d′ is not simply H–F; rather, it is the difference between the *z*-transforms of these two rates (cf. Macmillan & Creelman, 2005):

$$d^{\prime }\;=\;z(H)\;-\;z(F)$$

Considering discrimination studies (like our task; same–different matching task): hit rate H is defined as: proportion of different trials to which subject responded different = P(“different” | DIFFERENT) false alarm rate F is defined as proportion of same trials to which subject responded different = P(“different” |SAME).

**Figure 2.**
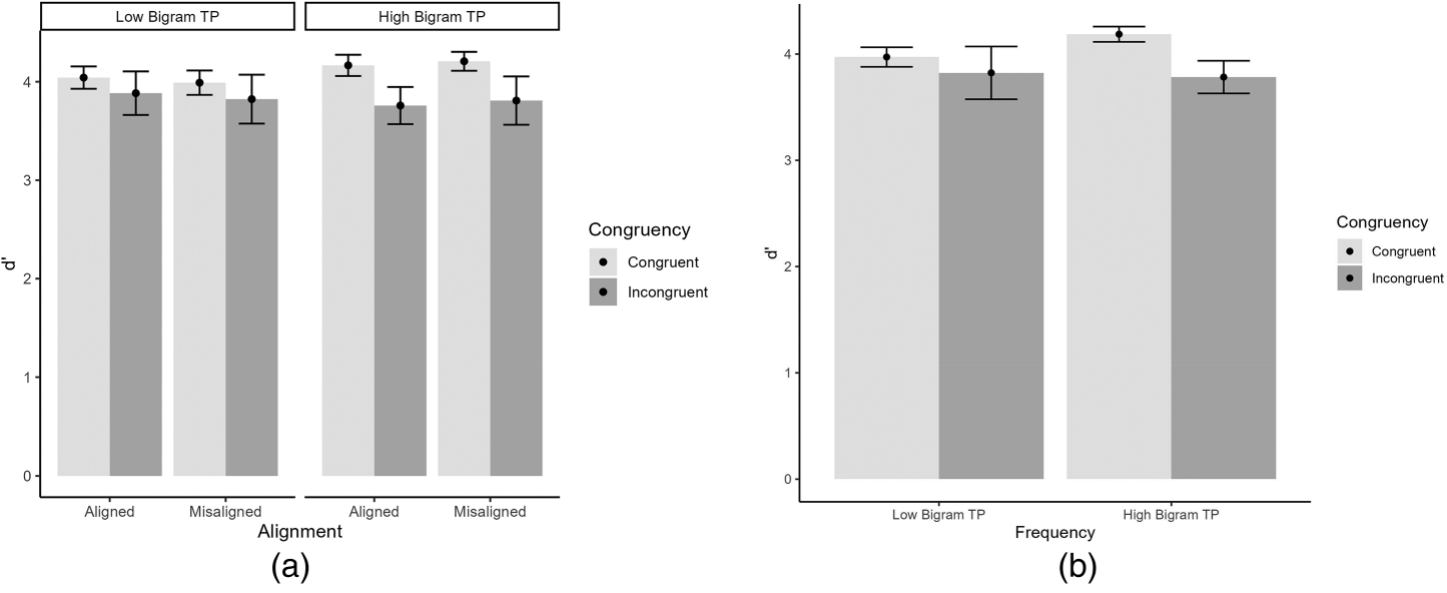
(a) d′ for low versus high bigram TP words across alignment and congruency. (b) Depiction of the interaction of TP and congruency.

A 2 (alignment: aligned, misaligned) × 2 (congruency: congruent, incongruent) × 2 (bigram TP: high, low) ANOVA performed on the sensitivity (d′) scores (cf. Figure 2) revealed a significant interaction of TP × Congruency, *F*(1, 38) = 4.05, *p *= .05, η_p_^2 ^= .10. We probed this interaction through post-hoc tests, looking at the effect of congruency for low and high transition probability words separately. There was a larger effect of congruency for high transition probability words (larger d′ for congruent than incongruent conditions, difference = 0.40, *SE *= 0.20; *t*(38) = 2.05, *p *= .048, than for lower bigram TP words, difference = 0.16, *SE *= 0.21; *t*(38) = 0.79, *p *= .433. The three-way interaction was not significant, *F *< 1. All other effects were not significant, including the interaction of alignment with congruency, *p*s > .15. To better capture whether these non-significant results reflect evidence against the existence of an effect, we consider inclusion of Bayes factors (BF_inc_), which reflect the extent to which the inclusion of an effect in a model increases its capability to explain the data (see above, and/or Lee & Wagenmakers, 2014). We find strong evidence against the inclusion of the Alignment × Congruency interaction (BF_inc _= 0.09) and moderate evidence against the inclusion of the three-way interaction (BF_inc _= 0.22), in line with the results of the frequentist analysis.

### Response-Time Analysis

**Figure 3.**
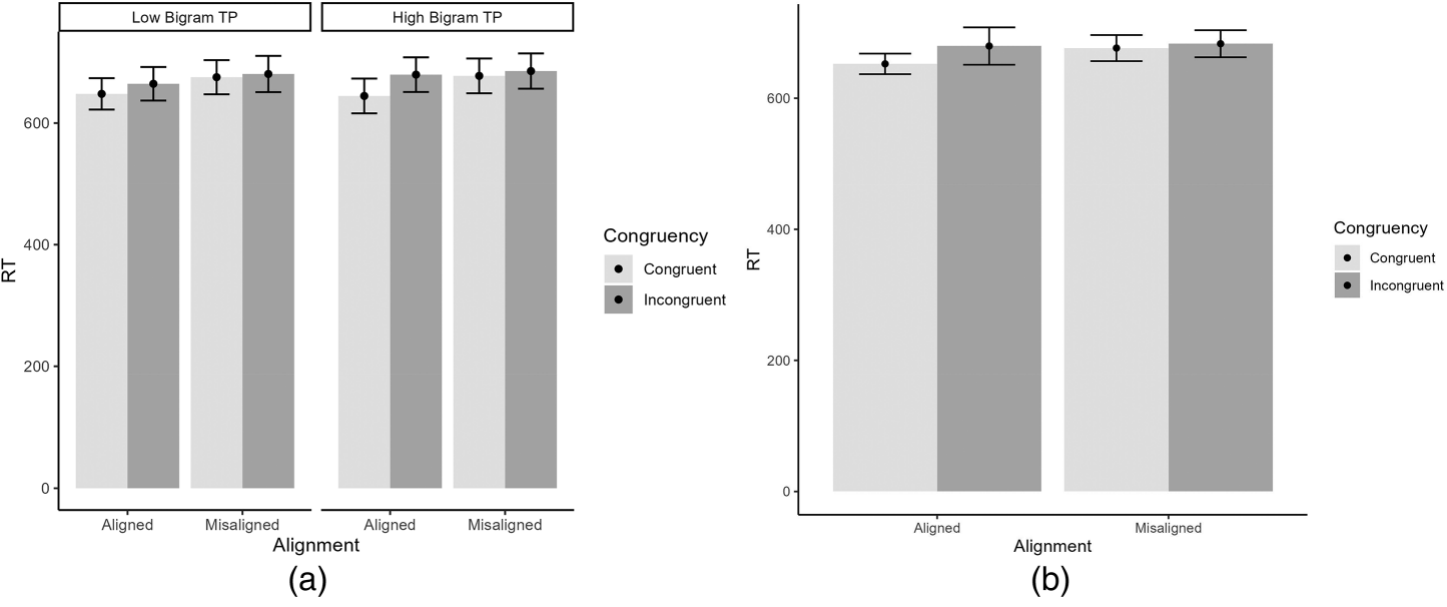
(a) RT for low versus high bigram TP words across alignment and congruency. (b) Depiction of the interaction of Alignment × Congruency (RT).

A 2 (alignment: aligned, misaligned) × 2 (congruency: congruent, incongruent) × 2 (bigram TP: high, low) ANOVA performed on RT data from correct trials (cf. Figure 3) revealed a main effect of congruency, *F*(1, 38) = 13.18, *p *< .001, η_p_^2 ^= .26, and an interaction of Alignment × Congruency, *F*(1, 38) = 7.08, *p *= .011, η_p_^2 ^= .16, indicative of holistic processing. The Alignment × Congruency interaction is described by significantly lower latencies for congruent over incongruent trials when the halves are aligned, difference = 25.82, *SE *= 6.07; *t*(38) = 4.25, *p *< .001, but no effect of (in)congruency when the halves are misaligned, difference = 6.62, *SE *= 5.40; *t*(38) = 1.23, *p *= .227. The three-way interaction was not significant, *F*(1, 38) = 1.73, *p *= .20, η_p_^2 ^= .04, and there is (congruent) moderate evidence against its inclusion (BF_inc _= 0.30). All other effects were not significant, *p*s > .08.

## Discussion

The visual system capitalizes on contextual information, such as the probabilities of stimuli co-occurrences, to accurately identify a word. We investigated one such contextual information, bigram TP, and its influence in word holistic processing. These TPs might act as a glue that binds together the two parts (syllables) of a four-letter disyllabic word and adds cohesiveness to each syllable. Defining holistic processing as the interaction between alignment and congruency, we found no evidence for a role of bigram TP in word holistic processing in the word composite task. In the RT analysis, we found the interaction of alignment and congruency indicative of holistic processing but no three-way interaction with bigram TP. In the sensitivity analysis, we found a significant interaction of bigram TP and congruency. For higher TP bigram words there was an effect of congruency (larger d′ for congruent than incongruent conditions). For lower TP bigram words there was no effect of congruency. However, we did not find the three-way interaction with bigram TP. The effect of congruency modulated by TP can be seen tentatively as a preliminary indication of the influence of bigram TP in holistic word processing.

We should note that in the RT analysis, we found a congruency effect and an interaction of alignment and congruency, both evidence of holistic processing, but no evidence that these effects interact with bigram TP. Nevertheless, in sensitivity analysis, we found a modulation of congruency by bigram TP. Although previous studies have reported a modulation of holistic processing using the RT measure, our finding of a modulation of holistic processing in sensitivity analysis, but not in RT analysis, was not unusual (e.g., Curby & Moerel, 2019).

What may explain the finding that high bigram TP amplifies the effect of congruency (but not low bigram TP)? When participants make identity judgments about the target halves of two composites, the irrelevant halves facilitate or interfere with target performance based on whether the identity relationship between the irrelevant halves is identical to (congruent) or different from (incongruent) that of the target halves (Jin et al., 2024). Considering congruent trials, the higher cohesiveness granted by high-frequency bigrams (cohesiveness of each syllable and cohesiveness between syllables) makes it easier to process simultaneously both parts of a word in the composite task reinforcing the facilitative effect of irrelevant halves and the holistic attentional strategy that develops through experience processing words.

The information required to identify a word is spatially distributed across the word. Holistic processing in the composite task depends on a tendency to attend to the visual stimuli as a whole: a holistic attentional strategy is optimal and thus develops through experience processing these stimuli. However, tentatively, it seems that bigram TP adds cohesiveness to words, thus linguistic sublexical factors may influence holistic processing. Another study showed also the influence of linguistic factors on holistic processing (Ventura et al., 2019). This study suggests that holistic processing of visual words is modulated by fast and automatic activation of lexical phonological representations.

In a future study, we will evaluate the role of trigram probability in word HP. In the word puro, <pur > corresponds to trigram 1, <uro > to trigram 2. Both trigrams span the two syllables and would strengthen the connections between the two syllables, maybe leading to a stronger cohesiveness of words than that offered by bigrams, making interference effects stronger. Indeed, high trigram TP would probably make the holistic processing stronger because the information from the second syllable would be more forcefully required (whereas when it is about bigram frequency, two out of the three bigrams contribute independently to the response—overlapping with the relevant and the irrelevant syllables).

## Appendix A

Words used in the experiment. English translation in brackets. Bigram probabilities (respectively for each word, the number and mean number of words that share bigrams and the frequency of their bigrams (both mean and summed). The words juta, buda and tejo do not appear in P-Pal lexical database.

**Table:**

Word1	Word 2	Word 3	Word 4	TP
caro (dear); 121; 40, 33; 110.3; 13346.52	pule (jump); 79; 26, 33; 4.93; 389.81	cale (shut up); 120; 40; 23.79; 2854.35	puro (pure);97; 32, 33; 9.78; 948.55	High
cora (blush); 116; 38.67; 180.04; 20885.0	vise (visa); 59; 19.67; 30.47; 1797.56	cose (sew); 75; 25; 120.02; 9001.34	vira (turn); 104; 34, 67; 123.86; 12881.65	High
cume (ridge); 56; 18, 67; 31.84; 1782.82	fora (outside); 89; 29.67; 151.56; 13489.01	cura (heal); 88; 29.33; 141.96; 12492.67	fome (hunger); 65; 21.67; 149.6; 9724.22	High
doca (dock); 97; 32.33; 30.33; 2942.48	pise (step); 65; 21.67; 15.61; 1014.58	dose (dosage); 64; 21.33; 49.95; 3196.6	pica (prick); 92; 30.67; 7.36; 677.2	High
fura (pierce); 92; 30.67; 133.32; 12265.68	belo (beautiful); 83; 27.67; 88.6; 7353.73	fulo (angry); 75; 25; 35.07; 2630.1	bera (bad); 85; 28.33; 155.32; 13201.82	High
gire (rotate); 70; 23.33; 2.96; 207.14	fula (angry); 87; 29; 32.55; 2831.46	gila (pumpkin gila); 74; 24.67; 37.88; 2803.46	fure (pierce); 74; 24.67; 6.97; 515.32	High
lusa (Portuguese); 55; 18.33; 21.3; 1171.66	meto (incase); 98; 32.67; 23.07; 2260.57	luto (grief); 80; 26.67; 20.43; 1634.03	mesa (table); 64; 21.33; 31.0; 1983.31	High
rima (rime); 78; 26; 21.89; 1706.53	fuso (spindle); 50; 16.67; 33.24; 1662.02	riso (laughter); 68; 22.67; 25.5; 1733.76	fuma (smokes); 69; 23 38.36; 2646.84	High
vice (vice); 56; 18.67; 24.59; 1376.87	faro (flair); 98; 32.67; 121.91; 11946.81	viro (I turn); 103; 34.33; 15.22; 1567.14	face (face); 56; 18.67; 22.32; 1249.69	High
cota (quota); 124; 41.33; 81.67; 10126.52	jure (swear); 62; 20.67; 9.52; 590.38	core (blush); 98; 32.67; 93.21; 9134.74	juta (jute)	High
cabo (cable); 82; 27.33; 31.98; 2622.49	tule (tulle); 71; 23.67; 15.4; 1093.53	cale (shut up); 120; 40; 23.78; 2854.35	tubo (tube); 46; 15.33; 19.54; 898.53	Low
cume (summit); 56; 18.67; 17.82; 1782.82	gabo (appreciate); 68; 22.67; 7.69; 523.16	cubo (cube); 44; 14.67; 9.65; 424.72	game (steal); 77; 25.67; 7.34; 565.54	Low
buda (budha);	mexo (I move); 51; 17; 20.12; 1026.1	buxo (boxwood); 26; 8.67; 6.7; 174.21	meda (rick); 88; 29.33; 41; 3606.89	Low
vime (wicker); 65; 21.67; 22.5; 1462.69	ruga (wrinkle); 82; 27.33; 6.14; 503.7	viga (beam); 80; 26.67; 17.55; 1404.0	rume (rumen); 68; 22.67; 23.61; 1605.66	Low
fila (row); 95; 31.67; 32.67; 3103.53	nego (I deny); 56; 18.67; 22.68; 1269.85	figo (fig); 74; 24.67; 21.12; 1563.23	nela (in her); 93; 31; 81.0; 7533.35	Low
fuga (escape); 74; 24.67; 6.13; 453.85	tome (take); 75; 25; 181.4; 9855.34	fume (smoke); 60; 20; 25.93; 1555.82	toga (toga); 81; 27; 25.14; 2036.09	Low
fula (angry); 87; 29; 32.55; 2831.46	tejo (tagus river)	fujo (I run away); 46; 15.33; 12.31; 566.39	tela (screen); 104; 34.67; 80.83; 8406.6	Low
liga (turns on); 74; 24.67; 8.52; 630.6	sumo (juice); 75; 25; 140.95; 10571.07	limo (slime); 67; 22.33; 126.56; 8479.6	suga (suck); 81; 27; 20.73; 1678.8	Low
lixo (garbage); 42; 14; 9.32; 391.25	puma (puma); 75; 25; 35.26; 2644.22	lima (lime); 68; 22.67; 26.18; 1780.3	puxo I pull); 43; 14.33; 5.77; 248.25	Low
luva (glove); 44; 14.67 21.79; 958.8	some (add); 77; 25.67; 118.23; 9103.62	lume (fire); 61; 20.33; 28.34; 1728.75	sova (thrashing); 64; 21.33; 35.61; 2279.44	Low

## Appendix B

All the trials for the quartet “caro, pule, cale, puro.”

**Table:**

Same–congruent	caro-caro
Different–congruent	caro-pule
Same–incongruent	caro-cale
Different–incongruent	caro-puro
Same–congruent	pule-pule
Different–congruent	pule-caro
Same–incongruent	pule-puro
Different–incongruent	pule-cale
Same–congruent	cale-cale
Different–congruent	cale-puro
Same–incongruent	cale-caro
Different–incongruent	cale-pule
Same–congruent	puro-puro
Different–congruent	puro-cale
Same–incongruent	puro-pule
Different–incongruent	puro-caro
